# Study on the Role of Schisandrin B in Ameliorating Hepatic Ischemia-Reperfusion Injury by Modulating Hepatocyte Autophagy

**DOI:** 10.5812/ijpr-157033

**Published:** 2025-04-13

**Authors:** Zhao Fei, Pan Haihang, Chen Xueyang, Shen Miao

**Affiliations:** 1 Center for General Practice Medicine, Department of Gastroenterology, Zhejiang Provincial People's Hospital (Affiliated People's Hospital), Hangzhou Medical College, Hangzhou Zhejiang, China

**Keywords:** Sch B, HIRI, Liver Injury, Autophagy, Damage Repair

## Abstract

**Background:**

Hepatic ischemia-reperfusion injury (HIRI) significantly affects the prognosis of liver surgery, such as hepatocellular carcinoma resection and liver transplantation. However, the pathogenesis of HIRI has not been fully elucidated, and prevention and treatment strategies remain challenging.

**Methods:**

A mouse model of HIRI was established, and schisandrin B (Sch B) was used to intervene in HIRI. The effect of Sch B on HIRI was assessed using hematoxylin and eosin (HE) staining, quantitative polymerase chain reaction (qPCR), Western blot (WB), immunohistochemistry, and enzyme-linked immunosorbent assay (ELISA).

**Results:**

The expression of alanine aminotransferase (ALT) and aspartate aminotransferase (AST) in the serum of the HIRI mouse model was significantly increased, indicating the successful construction of the HIRI mouse model. In the Sch B intervention group, serum ALT and AST levels were significantly decreased. Hematoxylin and eosin staining and qPCR results demonstrated that Sch B could reduce HIRI in mice. The qPCR and immunohistochemistry showed that Sch B reduced HIRI in mice by decreasing the expression of autophagy-related factors Beclin-1 and LC3-II.

**Conclusions:**

Additionally, qPCR and immunohistochemical results indicated that Sch B reduced HIRI by decreasing the expression of autophagy-related factors (Beclin-1 and LC3-II) and hepatocyte damage-related factors (caspase-3, caspase-9, and Bax), thereby reducing HIRI in mice. The results of electron microscopy, immunohistochemistry, and qPCR confirmed that Sch B could reduce autophagy and alleviate HIRI.

## 1. Background

Hepatic ischemia-reperfusion injury (HIRI) is a phenomenon in which tissue damage is aggravated or even irreversible damage occurs after restoring blood flow following hepatic ischemia, often occurring after hepatic cancer resection and liver transplantation. It has a severe adverse effect on the prognosis of liver surgery (1). The mechanism of HIRI has yet to be completely clarified, and prevention and control strategies remain challenging. Current research indicates that HIRI is primarily linked to intracellular calcium overload, excessive oxygen-free radical generation, and the secretion of numerous cytokines (2-6). Research on liver ischemia-reperfusion (IR) injury and screening of protective drugs rely heavily on the 70% IR animal model, which has the advantages of less injury and a higher survival rate than the total ischemia model. The pathological mechanism of HIRI is complex, involving autophagy, mitochondrial damage, oxidative stress imbalance, abnormal cell death, immune cell overactivation, intracellular inflammatory disorders, and other complex events (7, 8). Despite serious clinical limitations, available antagonists and specific treatment options are still lacking.

Several studies have explored treatments for HIRI, such as 5-aminolevulinic acid and CHIL3/CHI3L1, which can help liver metabolism and reduce IR injury (9). Nano-cerium oxide (NPs), as a representative nano-antioxidant, has been found to effectively alleviate the clinical symptoms of hepatic IRI by clearing reactive oxygen species (ROS) and inhibiting the activation of Kupffer cells and mononuclear/macrophages (10). Gut microbial-derived glutamine alleviates liver ischemia/reperfusion injury through macrophage metabolic reprogramming (11). Dendritic cell-mediated regulation of liver IR injury and liver transplant rejection has also been studied (12). Administration of SerpinB3 protects the liver from IR injury (13). Nepetoidin B alleviates HIRI by regulating the MKP5 and JNK/P38 pathways (14).

Research on the prevention and treatment of liver IR injury with traditional Chinese medicine is still lacking. schisandrin B (Sch B) has various functions, including antioxidant, anti-inflammatory, cardioprotective, and neuroprotective properties. The Sch B has been documented as an effective drug for treating several diseases by targeting signaling pathways such as nuclear factor erythroid 2-related factor 2 (Nrf2)-ARE and TGF-β/Smad (15, 16). There is no relevant study on whether Sch B has a therapeutic effect on HIRI. Recently, autophagy, as a new mode of cell death, has received increasing attention from scholars. Autophagy encompasses several distinct processes, including macroautophagy, microautophagy, and chaperone-mediated autophagy, with macroautophagy being the most thoroughly investigated. During macroautophagy, cytoplasmic constituents, intracellular organelles, and proteins are sequestered within autophagic vesicles. These vesicles, derived from the ribosome-free regions of the rough endoplasmic reticulum's double-layer membrane, are transported to the lysosome. Upon fusion, they form autophagic lysosomes, which degrade the enclosed materials to meet the cell's metabolic needs and facilitate organelle renewal (6, 17-19). At the physiological level, autophagy can clear intracellular stale organelles. However, the level of autophagy during IR injury far exceeds the physiological level. Excessive activation of autophagy markedly elevates lysosomal activity, culminating in irreversible cellular demise attributable to the disproportionate degradation of vital proteins and organelles (1, 20-24). Therefore, autophagy modulation has become one of the most essential tools for combating HIRI.

Clinicians do not have a specific drug for treating HIRI, making it significant to strengthen research on new drugs effective against this condition. Chinese medicine is popular domestically and internationally due to its stable effect and mild medicinal properties. So far, few studies have focused on effective Chinese medicines for IR injury. Most Chinese medicine compound preparations for IR, such as Jiaweizhu Tang and Sijunzi Tang, contain essential Chinese medicine ingredients like *Schisandra chinensis*, hawthorn, and cassia seeds, with *Sch. chinensis* being widely used. *Sch. chinensis* is the dried mature fruit of *Sch. chinensis* or Huazhong *Schisandra* of the Magnoliaceae family and is a traditional tonic Chinese herb. Fructus schizandra contains volatile oil, lignin, polysaccharides, organic acids, and other chemical components. Modern pharmacological research indicates that Fructus schizandra exhibits hepatoprotective, antioxidant, and anti-tumor properties, among other effects (25, 26).

*Schisandra chinensis* extracts contain various active components, with lignans being the main bioactive components. Lignans include *Sch. chinensis* ester A, Sch B, schisandrin methylin, Sch B, schisandrin A, and Sch B, among others (27). The molecular formula of Sc hB is C23H28O6, and the molecular weight is 400.46. Its chemical structure contains the biphenyl skeleton, a complex organic compound. Current studies have shown that Sch B has antioxidant, anti-inflammatory, metabolic regulation, anti-cancer, and tissue protection effects (28). More importantly, Sch B has the effect of scavenging free radicals and inhibiting oxidative stress, which can effectively reduce heart, brain, and kidney damage caused by IR (29-31).

In recent years, Sch B has attracted attention in drug research and development due to its various biological activities and potential medicinal value. Researchers are exploring its potential as an antioxidant, anti-tumor, anti-inflammatory agent, and more, conducting numerous basic and clinical studies. As an active ingredient extracted from natural plants, the study of Sch B has also promoted the development of the field of natural products. Through in-depth study, Sch B's various biological activities have been revealed, providing a scientific basis for its application in medicine, health products, and other fields. In summary, Sch B is a natural product with various biological activities and potential medicinal value. With ongoing research, its application prospects in drug development, natural product research, and other fields are expected to broaden. However, the role of this compound in HIRI remains unclear (27). Therefore, we hypothesize that Sch B is biologically active against HIRI. However, its effects and specific pharmacological mechanisms require further study.

## 2. Objectives

The objective of this study was to examine the effects of Sch B on a hepatic injury model induced by HIRI. The HE and biochemical analyses verified that the hepatic IR liver injury model was constructed successfully. The expression levels of cell injury markers, oxidative stress indicators, autophagy-related factors, and other pertinent variables were assessed using quantitative PCR (qPCR) and Western blot (WB) techniques. Subsequently, the impact of Sch B on a HIRI liver injury model was investigated. A series of experiments were conducted to elucidate the potential mechanisms of action of Sch B in HIRI, with a particular focus on its regulatory effects on autophagy-related factors.

## 3. Methods

### 3.1. Animal Models and Hepatic Ischemia-Reperfusion Surgery

Liaoning Changsheng Biotechnology Co., Ltd. provided 8-week-old male C57BL/6J mice (20 - 25 g) with license number SCXK (Liao) 2020-0001. The study was conducted under a 12-hour light-dark cycle, with free access to food and water, and housed in a standard pathogen-free (SPF) chamber. All animal experiments conducted in this study were reviewed and approved by the Animal Care and Use Committee of Zhejiang Provincial People's Hospital, China. These experiments followed REACH guidelines and relevant regulatory frameworks, including the UK's animals (scientific procedures) act 1986, EU directive 2010/63/EU, and the guidelines for the care and use of laboratory animals published by the US National Research Council.

Schisandrin B was dissolved in 0.5% sodium carboxymethylcellulose (CMC-Na) (61281-37-6, Herbpurify, China). The mice were randomly divided into six groups, each consisting of six individuals: Sham + vehicle, sham + Sch B, IR + vehicle, IR + Sch B, IR + Rap, and IR + Rap + Sch B. Schisandrin B (30 mg/kg) and rapamycin (2.0 mg/kg) were administered daily via intragastric administration one week before surgery, and 0.5% CMC-Na solution was given starting one week before surgery. Experimental mice fasted for 12 hours before surgery and were deprived of water 2 hours before surgery. Mice were anesthetized by intraperitoneal injection of 1% pentobarbital sodium at a dose of 50 mg/kg.

After the mice lost consciousness, the abdominal hair was removed, the abdomen was fully exposed, and the limbs of the mice were fixed with adhesive tape while the mice were supine on a foam board. The surgical site was disinfected with aner iodine solution, and the abdominal cavity of the mice was carefully incised to avoid damage to the xiphoid process. The liver and portal of the mice were carefully but fully exposed with sterile cotton swabs, and the hepatic artery and portal vein of the mice were occluded with non-invasive vascular clips, rendering the left and middle lobes of the liver ischemic. After vessel occlusion, the ischemic liver lobe changed from bright red to light red, especially at the liver edge, while the remaining liver lobe without vessel occlusion remained bright red. Fluid was replenished by intraperitoneal injection of sterile saline, and a small piece of gauze slightly dampened with sterile saline was placed over the abdominal cavity opening. The mice were then moved to a warm place and kept warm, with continuous monitoring of their state.

After 90 minutes, the non-invasive vessel clamp was carefully removed to restore blood flow to the ischemic liver, and the ischemic liver gradually turned red within approximately 30 seconds. The abdominal incision was sutured, and 0.2 mL of normal saline was injected into the abdomen again to replace the body fluid and blood lost after laparotomy. The mice in the sham operation group were only supplemented with normal saline without any other treatment after laparotomy, and the abdominal cavity was closed after 45 minutes. After surgery, the mice were placed on a heat blanket for six hours to regulate body temperature and monitor vital signs. The mice were euthanized before samples were collected (32). All animals were anesthetized with 3% isoflurane and euthanized by intravenous injection of pentobarbital sodium (150 mg/kg). All procedures were consistently executed by the same experimenter.

### 3.2. Liver Function Assessment

The Chemray 240 biochemistry analyzer (Shenzhen Radiology Life Technology, China) was used to measure serum alanine aminotransferase (ALT) and aspartate aminotransferase (AST) levels post-reperfusion.

### 3.3. Histopathological Examination

Tissue samples were fixed overnight in 4% paraformaldehyde, embedded in paraffin, and sectioned into serial slices of 4 μm thickness. Subsequent hematoxylin and eosin (HE) staining of the liver tissue sections was performed, followed by histological examination.

### 3.4. Enzyme-Linked Immunosorbent Assay

The ELISA kits were used to measure serum cytokine concentrations, including tumor necrosis factor-alpha (TNF-α), interleukin-1 (IL-1), and interleukin-6 (IL-6). The ELISA kits used were TNF-α (COIBO BIO, CB10851-Mu), IL-1 (COIBO BIO, CB10160-Mu), and IL-6 (COIBO BIO, CB10187-Mu).

### 3.5. Immunohistochemically Staining

For staining, tissue samples were fixed overnight in 4% paraformaldehyde, embedded in paraffin, and sectioned serially at a thickness of 4 μm. For immunohistochemical staining, the sections underwent deparaffinization, hydration, and antigen retrieval, and were incubated overnight at 4°C with primary antibodies, specifically Beclin-1 (1:100, A7353, Abclonal) and LC3-II (1:100, A21695, Abclonal). Following two washes with phosphate-buffered saline (PBS), the samples were incubated with a 1:10,000 dilution of horseradish peroxidase (HRP)-conjugated secondary antibody (AS014, Abclonal) for one hour at room temperature. Subsequently, the sections were visualized using 3,3'-diaminobenzidine (DAB) (P0202, Beyotime) and counterstained with hematoxylin.

### 3.6. Quantitative Polymerase Chain Reaction Analysis

Total RNA was extracted from mouse liver tissue using the TRIzol reagent (EP013, ELK Biotechnology). The synthesis of first-strand complementary DNA (cDNA) was subsequently carried out using the EntiLink^TM^ 1st Strand cDNA synthesis kit, inclusive of the gDNA Eraser (EQ003, ELK Biotechnology), in accordance with the manufacturer's protocol. The qPCR was performed using the SYBR RT-PCR kit (Takara, Tokyo, Japan) on the step one real-time PCR system (EQ001, ELK Biotechnology). Reverse transcription was performed, and qPCR was conducted with the SYBR RT-PCR kit (Takara, Tokyo, Japan) on the step one real-time PCR system. Normalization was conducted based on glyceraldehyde 3-phosphate dehydrogenase (GAPDH) relative expression. Primer sequences are listed in Table 1. 

**Table 1.** List of Quantitative Polymerase Chain Reaction Primers

Genes	Forward (5’ to 3’)	Reverse (5’ to 3’)
**Caspase-3 **	GGGGAGCTTGGAACGCTAAG	CCGTACCAGAGCGAGATGAC
**Caspase-9 **	CAGTCCCTCCTTCTCAGGGTTG	TGCATCGCCAAAGGGAAAGA
**Bax**	AAACTGGTGCTCAAGGCCC	GCCTCAGCCCATCTTCTTCC
**HO-1**	TGCTAGCCTGGTGCAAGATAC	GCTAGGGACCCCAAAAGC
**Nrf2**	ATCTCCTAGTTCTCCGCTGC	CAAATCCATGTCCTGCTGGG
**Beclin-1**	AGGCTAACTCAGGAGAGGAGC	GCCCTCAGTGCCTCATCATTA
**MAP1LC3B**	ACAAGGGAAGTGATCGTCGC	TCGCTCTATAATCACTGGGATCT
**GAPDH**	CTTCTCCTGCAGCCTCGT	CCAATACGGCCAAATCTTGAGG

Abbreviation: GAPDH, glyceraldehyde 3-phosphate dehydrogenase; MAP1LC3B, microtubule-associated protein 1 light chain 3 beta; HO-1, heme oxygenase-1; Nrf2, nuclear factor erythroid 2-related factor 2.

### 3.7. TEM Analysis

Transmission electron microscopy was used to detect autophagosomes. Liver tissues were fixed with 2.5% glutaraldehyde and 0.1 mol/L sodium acetate at 44°C for 2 - 4 hours, followed by dehydration, embedding, sectioning, and staining. A minimum of three randomly selected regions were evaluated through a blinded ultrastructural assessment technique, utilizing an HT7700 transmission electron microscope (HITACHI, Tokyo, Japan).

### 3.8. Immunoblotting

The quantification of target proteins in liver samples was conducted through WB analysis, employing established standard methodologies. Each sample comprised 30 μg of total protein. The antibodies used were Bax (1:1000, A15646, Abclonal, China), caspase-3 (1:1000, A19654, Abclonal, China), caspase-9 (1:1000, A11451, Abclonal, China), Beclin-1 (1:800, A7353, Abclonal, China), LC3A/B (1:1000, AF5402, Affinity, China), and β-actin (1:5000, AC038, Abclonal, China). Data were collected using the ChemiDoc XRS + chemiluminescent imaging system, and immunoblot bands were densitometrically analyzed using ImageJ (NIH, Bethesda, MD, USA).

### 3.9. Statistical Analysis

Qualitative data were collected from a minimum of three independent experiments. Tukey’s post hoc test was conducted following one-way analysis of variance (ANOVA) to evaluate differences between groups, with a significance threshold set at P < 0.05. Data analysis was performed using GraphPad Prism 9 (San Diego, CA, USA) (33).

## 4. Results

### 4.1. Schisandrin B Protects IR-induced Liver Injury

**Figure 1.**
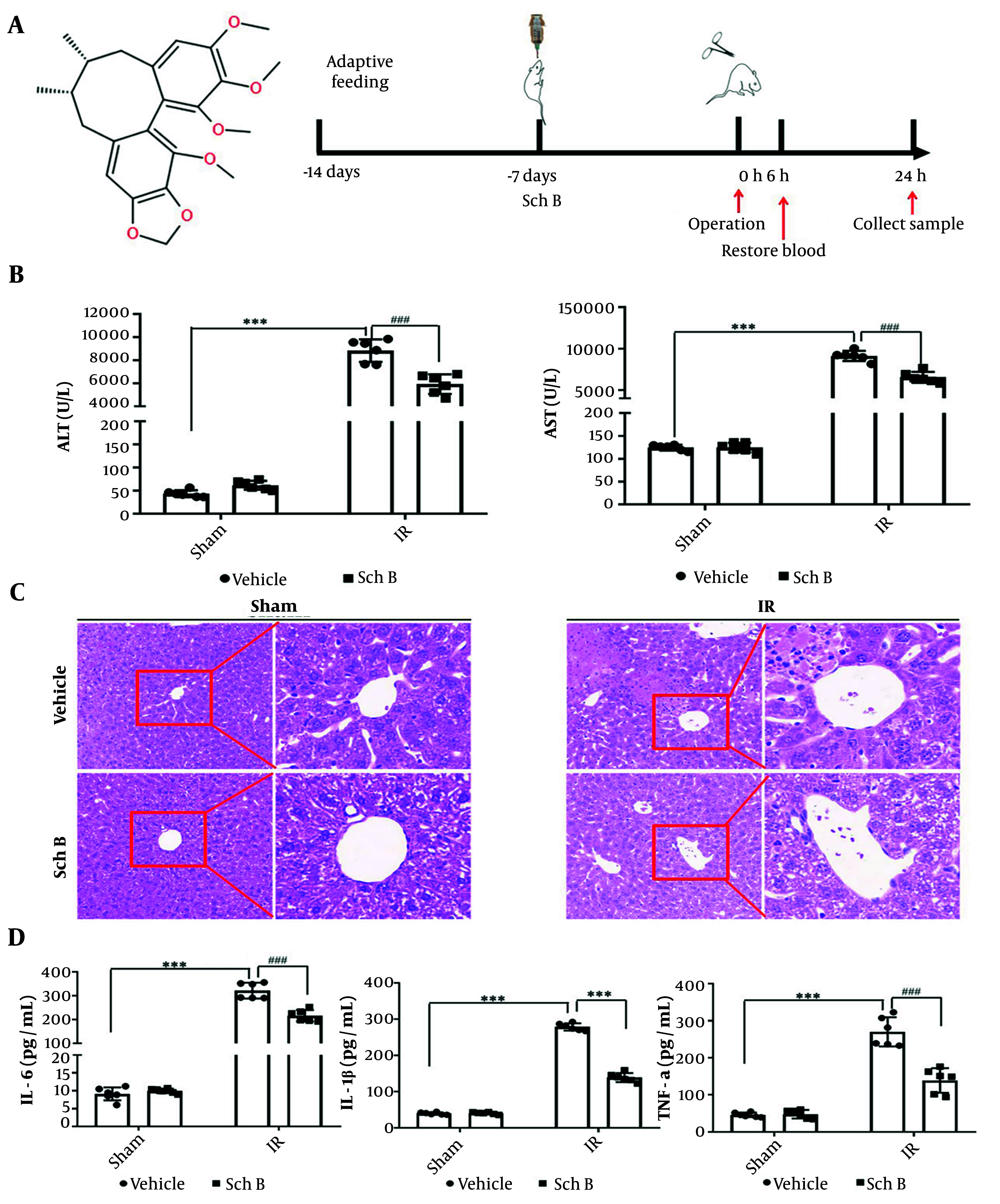
Protective effect of schisandrin B (Sch B) against liver ischemia-reperfusion (IR) injury in mice. A, Schematic diagram of the chemical structure and animal experimental procedure of Sch B; B, serum alanine aminotransferase (ALT) and aspartate aminotransferase (AST) levels (n = 6), The results of one-way analysis of variance (ANOVA) for ALT and AST were F (DFn, DFd) = F (3, 20) = 274.5, P < 0.0001 and F (3, 20) = 624.6, P < 0.0001, respectively; C, representative HE-stained liver sections (200X or 400X) under digital microscope; D, serum interleukin-1β (IL-1β), IL-6, TNF-a levels (n = 6). The results of one-way ANOVA for IL-1β, IL-6, and TNF-a were F (DFn, DFd) = F (3, 20) = 343.7, P < 0.0001, F (3, 20) = 1098, P < 0.0001, and F (3, 20) = 94.50, P < 0.0001, respectively. Data are mean ± standard deviation. Ischemia-reperfusion + Vehicle vs. Sham + Vehicle. *** P < 0.001; IR + Sch B vs. IR + Vehicle, ### P < 0.001.

Schisandrin B derived from the fruit of *Sch. chinensis*, contains the highest concentration of bifycloctene lignans among traditional Chinese medicines. The molecular formula is C23H28O6. Figure 1A illustrates the chemical structure. One week prior to IR surgery, mice received a daily gavage of either 30 mg/kg Sch B or an equivalent volume of 0.5% CMC-Na solution to assess liver injury post-IR (Figure 1A). The results demonstrated that, compared to the sham + vehicle group, the sham + Sch B group exhibited no significant alterations in ALT and AST levels, suggesting an absence of notable hepatotoxicity. Conversely, the IR + vehicle group showed a marked increase in ALT and AST levels, confirming the successful establishment of the IR damage model. Furthermore, ALT and AST levels were significantly reduced in the IR + Sch B group relative to the IR + vehicle group (Figure 1B). 

The histopathologic evaluation of liver tissue stained by HE showed that cytoplasmic concentration disturbance and cell structure destruction, including necrosis, verification, and fragmentation of fusion plates, occurred 24 hours after operation in the IR + vehicle group. In the Sch B + vehicle treatment group, necrosis was significantly reduced, indicating that the IR injury model was successfully established, and the IR + Sch B group significantly reduced liver injury (Figure 1C). 

The ELISA results indicated that, compared to the sham group, the levels of inflammation-related factors interleukin-1β (IL-1β), IL-6, and TNF-α did not exhibit significant changes in the sham + Sch B group. Conversely, the IR + vehicle group demonstrated a significant increase in the levels of IL-1β, IL-6, and TNF-α. Notably, the IR + Sch B group showed a reduction in these inflammation-related factors (Figure 1D). These findings suggest that Sch B exerts a protective effect against IR-induced liver injury.

### 4.2. Schisandrin B Protects Against IR-induced Hepatocyte Death

**Figure 2.**
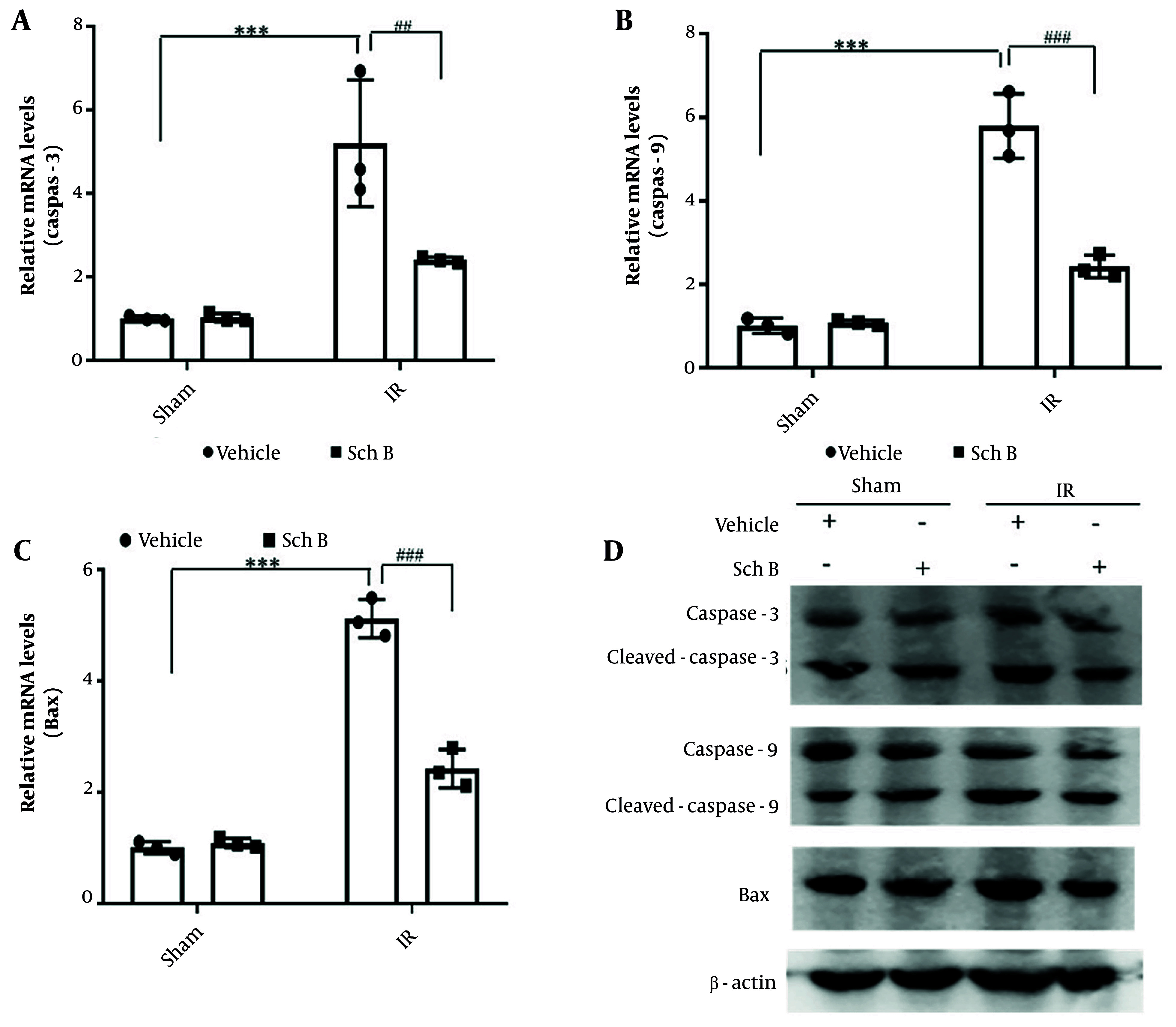
Effect of schisandrin B (Sch B) on hepatocyte injury in mice. A - C, Quantitative polymerase chain reaction (qPCR) detection of mRNA levels of caspase-3, caspase-9, and Bax in liver tissues (n = 3), The results of one-way analysis of variance (ANOVA) for caspase-3, caspase-9, and Bax were F (DFn, DFd) = F (3, 8) = 20.08, P < 0.0004, F (3, 8) = 85.18, P < 0.0001, and F (3, 8) = 172.9, P < 0.0001, respectively; D, immunoblotting for protein levels of caspase-3, caspase-9, and Bax in liver tissues. Data are mean ± standard deviation. IR + Vehicle compared with Sham + Vehicle. *** P < 0.001; IR + Sch B compared with IR + Vehicle, ## P < 0.01, ### P < 0.001.

Liver tissue injury was evaluated by WB and qPCR. The results indicated that, compared to the sham + vehicle operation group, the mRNA and protein levels of caspase-3, caspase-9, and Bax in the sham + Sch B group exhibited no significant alterations. Conversely, the mRNA and protein levels of caspase-3, caspase-9, and Bax were markedly elevated in the IR + vehicle group. Notably, the IR + Sch B group demonstrated a significant reduction in the mRNA and protein levels of caspase-3, caspase-9, and Bax (Figure 2A - D). 

### 4.3. Schisandrin B May Act Mainly Through the Autophagy Pathway of Liver Cells

**Figure 3.**
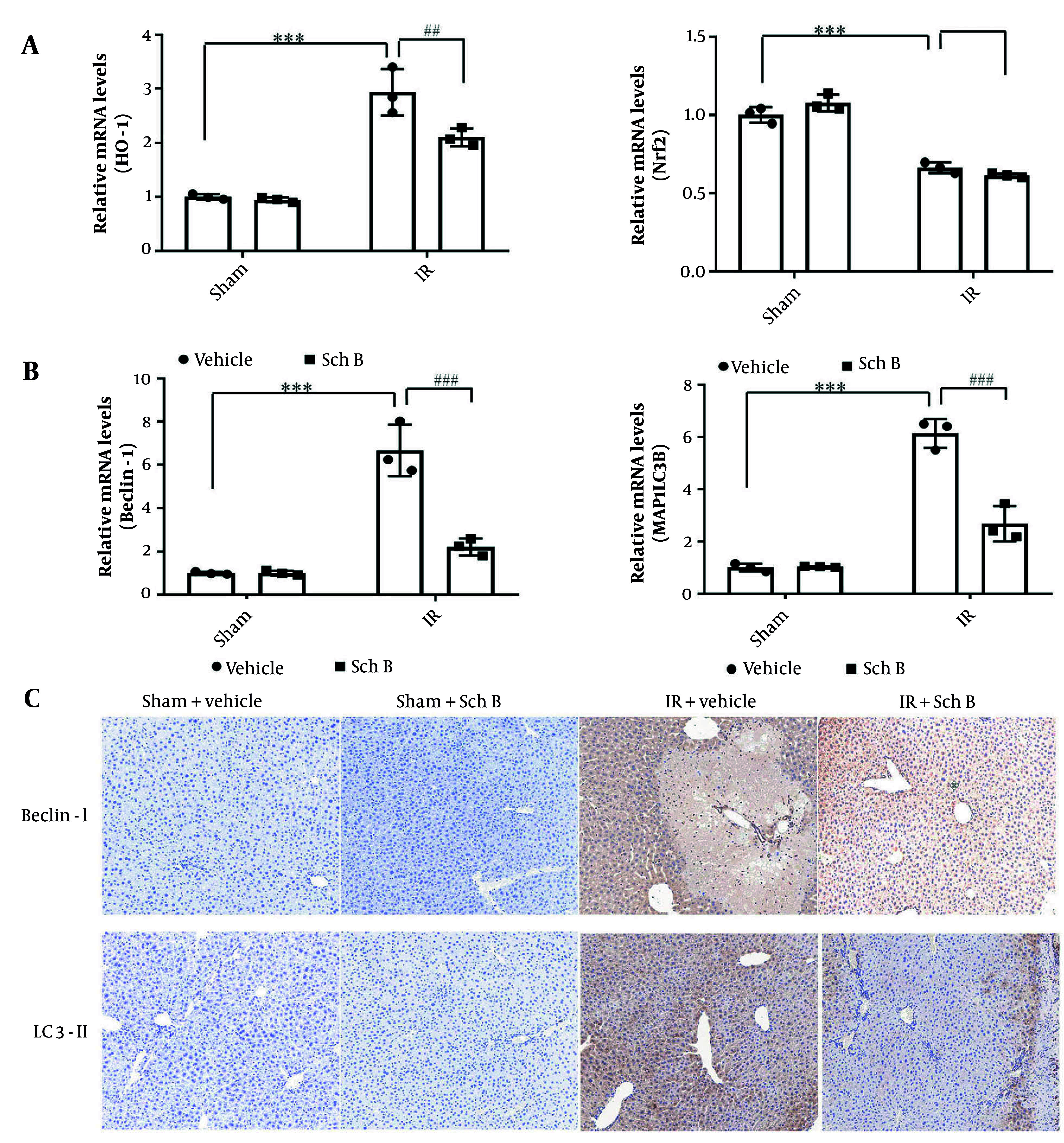
Effects of schisandrin B (Sch B) on oxidative stress and cellular autophagy in mice. A, Quantitative polymerase chain reaction (qPCR) detection of mRNA levels of oxidative stress-related factors HO-1 and nuclear factor erythroid 2-related factor 2 (Nrf2) in liver tissues (n = 3), The results of one-way analysis of variance (ANOVA) for HO-1 and Nrf2 were F (DFn, DFd) = F (3, 8) = 50.87, P < 0.0001 and F (3, 8) = 99.61, P < 0.0001, respectively; B, quantitative polymerase chain reaction detection of mRNA levels of cellular autophagy-related factors Beclin-1, microtubule-associated protein 1 light chain 3 beta (MAP1LC3B) in liver tissues (n = 3), The results of one-way ANOVA for Beclin-1 and MAP1LC3B were F (DFn, DFd) = F (3, 8) = 54.55, P < 0.0001 and F (3, 8) = 88.96, P < 0.0001, respectively; C, immunohistochemical detection of cellular autophagy-related factors Beclin-1, LC3-II (200×). Data are mean ± standard deviation. IR + Vehicle vs Sham + Vehicle. *** P < 0.001; IR + Sch B vs IR + Vehicle. ## P < 0.01, ### P < 0.001; Abbreviation: n.s, no significant difference.

Changes in oxidative stress and autophagy in liver tissue were evaluated using qPCR. The qPCR analysis showed that mRNA expression levels of oxidative stress-related genes heme oxygenase-1 (HO-1) and Nrf2 in the sham + Sch B group had no significant changes compared with the sham + vehicle group. Conversely, the mRNA level of HO-1 was significantly increased in the IR + vehicle group, while the mRNA level of Nrf2 was significantly decreased (Figure 3A). Additionally, mRNA levels of HO-1 were significantly reduced in the IR + Sch B group compared to the IR + vehicle group, while mRNA levels of Nrf2 showed no difference (Figure 3A). 

The qPCR analysis showed that mRNA expression levels of Beclin-1 and microtubule-associated protein 1 light chain 3 beta (MAP1LC3B), essential genes for autophagy in hepatocytes, in the sham + Sch B group had no significant changes compared with the sham operation group. In contrast, Beclin-1 and MAP1LC3B mRNA levels were significantly increased in the IR + vehicle group by about 1.5 times (Figure 3B). Notably, Beclin-1 and MAP1LC3B mRNA levels were significantly reduced in the IR + Sch B group, about three times as much as in the IR + vehicle group (Figure 3B). 

Immunohistochemical and qPCR analyses revealed that, compared to the sham + Sch B group, the expression levels of Beclin-1 and LC3-II proteins in hepatocytes of the sham + Sch B group did not exhibit significant alterations. Conversely, the IR + vehicle group demonstrated a marked upregulation of Beclin-1 and LC3-II proteins (Figure 3C). Furthermore, the IR + Sch B group showed significantly reduced levels of Beclin-1 and LC3-II proteins compared to the IR + vehicle group (Figure 3C). These findings indicate that Sch B may mitigate HIRI by inhibiting autophagy.

### 4.4. Schisandrin B Protects Against Hepatic IRI by Regulating Autophagy

**Figure 4.**
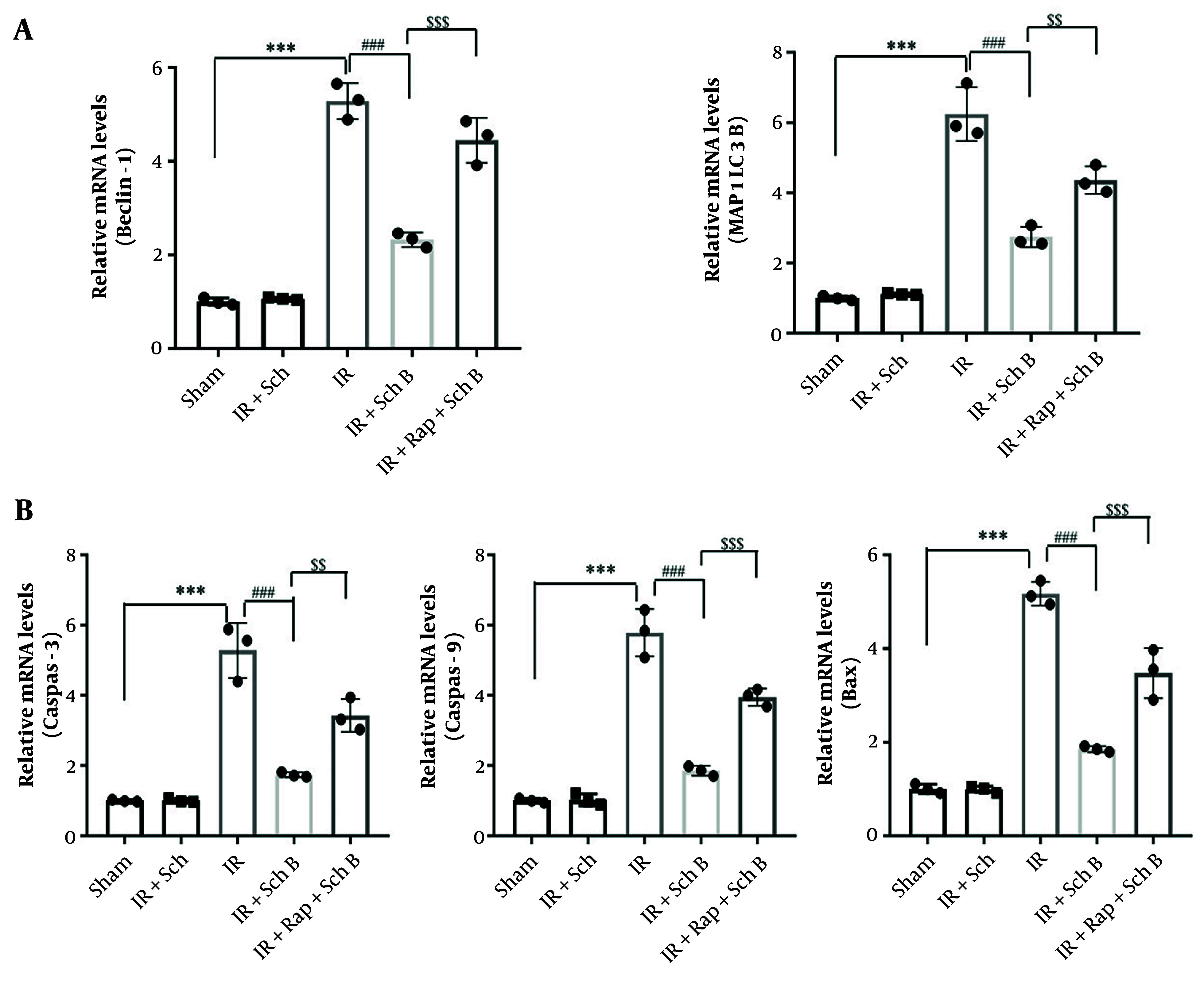
Schisandrin B (Sch B) reduces liver ischemia-reperfusion (IR) injury by inhibiting cellular autophagy. A, Quantitative polymerase chain reaction (qPCR) detection of mRNA levels of cellular autophagy-related factors Beclin-1, microtubule-associated protein 1 light chain 3 beta (MAP1LC3B) in liver tissues (n = 3), The results of one-way analysis of variance (ANOVA) for Beclin-1 and MAP1LC3B were F (DFn, DFd) = F (4, 10) = 141.1, P < 0.0001 and F (4, 10) = 90.20, P < 0.0001, respectively; B, quantitative polymerase chain reaction detection of mRNA levels of caspase-3, caspase-9, and Bax in liver tissues (n = 3), The results of one-way ANOVA for caspase-3, caspase-9, and Bax were F (DFn, DFd) = F (4, 10) = 61.05, P < 0.0001, F (4, 10) = 114.8, P < 0.0001, and F (4, 10) = 131.6, P < 0.0001, respectively. Data are mean ± standard deviation. P < 0.001 for IR + Vehicle vs. Sham + Vehicle. *** P < 0.001; P < 0.001 for IR + Sch B vs. IR + Vehicle, ### P < 0.001; $$ P < 0.01 and $$$ P < 0.001 for IR + Rap + Sch B vs. IR + Sch B.

An in vivo autophagy activation model was established by intraperitoneal injection of rapamycin. The expression levels of autophagy-related genes Beclin-1 and LC3-II were analyzed using qPCR. The mRNA levels of Beclin-1 and MAP1LC3B were significantly reduced in the IR + Sch B group compared to the IR + vehicle group (Figure 4A and B). Conversely, the mRNA levels of Beclin-1 and MAP1LC3B in the IR + Sch B + Rap group were significantly elevated compared to those in the IR + Sch B group (Figure 4A and B).

The expression levels of damage-related genes (caspase-3, caspase-9, Bax) were quantified using qPCR. Compared to the IR + vehicle group, the mRNA expression levels of caspase-3, caspase-9, and Bax were significantly reduced in the IR + Sch B group. Similarly, the mRNA levels of these genes were also significantly decreased in the IR + Sch B + Rap group. In contrast, when compared to the IR + Sch B group, the mRNA levels of Beclin-1 and MAP1LC3B were significantly elevated in the IR + Sch B + Rap group, accompanied by a significant increase in the expression of injury-related genes (caspase-3, caspase-9, Bax) (Figure 4A and B).

**Figure 5.**
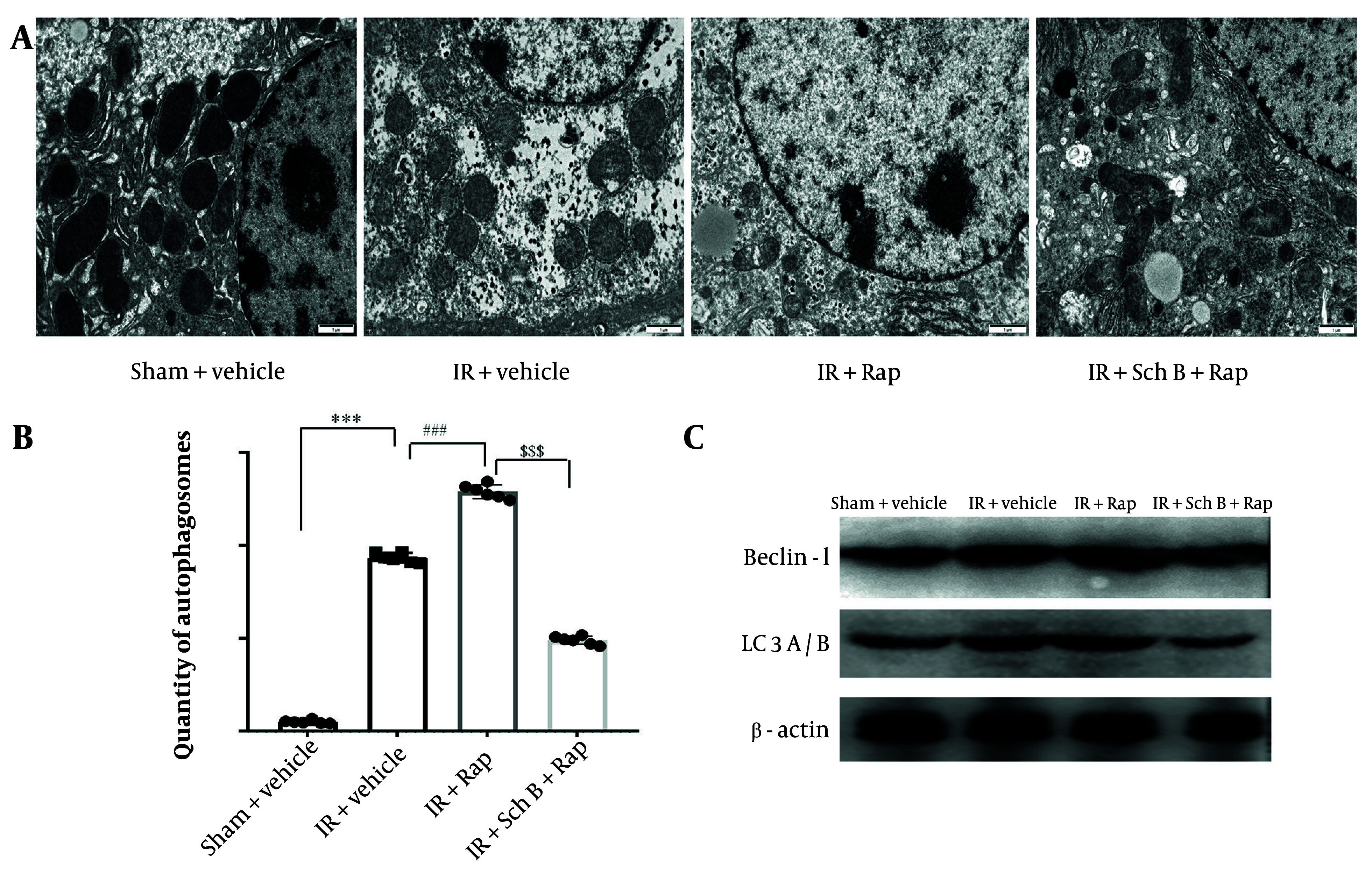
Effect of schisandrin B (Sch B) on cellular autophagy. A and B, The formation of autophagic vesicles in different groups was detected by electron microscopy, and statistical analysis was done, The results of one-way analysis of variance (ANOVA) were F (DFn, DFd) = F (3, 20) = 2628, P < 0.0001; C, immunoblotting was performed to detect the protein levels of Beclin-1 and LC3A/B in liver tissues. Data are mean ± standard deviation. Ischemia-reperfusion vs. Sham. *** P < 0.001; IR + Sch B vs. IR, ### P < 0.001; IR + Rap + Sch B vs. IR + Sch B, $$$ P < 0.001.

The formation of autophagic vesicles in each experimental group was examined using electron microscopy. The results indicated a significant increase in autophagic vesicles in the IR + Rap group compared to the IR group (Figure 5A and B). Conversely, a significant reduction in autophagic vesicles was observed in the IR + Sch B + Rap group relative to the IR + Rap group (Figure 5A and B).

The expression levels of key autophagy proteins in each experimental group were assessed using WB analysis. Notably, the levels of Beclin-1 and LC3-II were significantly elevated in the IR + Rap group compared to the IR group (Figure 5C). Conversely, the levels of Beclin-1 and LC3-II were significantly decreased in the IR + Sch B + Rap group relative to the IR + Rap group (Figure 5C). These results suggest that Sch B acts on hepatic IR by inhibiting autophagy.

## 5. Discussion

In this study, it was found that Sch B may ameliorate HIRI by regulating the expression of autophagy-related genes. Studies have shown Sch B as a potential drug for clinical transformation, such as promoting human umbilical cord mesenchymal stem cell differentiation into the liver (34), providing a protective effect against human hepatocellular toxicity induced by acetaminophen (15), alleviating CCl4-induced liver fibrosis in rats by regulating Nrf2-ARE and TGF-β/Smad signaling pathways (15, 16), and reducing inflammation, oxidative stress, and apoptosis induced by traumatic spinal cord injury by inhibiting the p53 signaling pathway (35). However, there have been no studies on the improvement of Sch B on the prognosis of patients undergoing liver surgery or transplantation. This study provides a basis for further research on the potential of Sch B to improve the prognosis of patients undergoing liver surgery or transplantation.

Many related genes regulate the autophagy process. Among them, the expression level of LC3 is considered a marker of autophagy level and can reflect the autophagy level. After autophagy is induced, LC3-I in the cytosol is cleaved and covalently binds phosphatidylethanolamine to form LC3-II localized at the autophagosome membrane. LC3-II is then specifically expressed on autophagosomes. Additionally, Beclin-1 is recognized as an essential regulatory gene in autophagy. Current studies have found mitochondrial dysfunction and autophagy in hepatic ischemia/reperfusion injury (36). However, the effect of autophagy on hepatic ischemia/reperfusion injury by regulating autophagy has yielded different results. Some studies have found that activation of autophagy can improve hepatic ischemia/reperfusion injury. For example, nobiletin improves liver IR injury by activating SIRT-1/FOXO3A-mediated autophagy and mitochondrial biogenesis (37). Other studies have found that inhibition of autophagy can improve liver IR injury. KLF6 alleviates HIRI by inhibiting autophagy (38). Proanthocyanidins inhibit autophagy and apoptosis through the PPAR-α/PGC1-α signaling pathway to reduce liver IR injury (39). Overexpression of seipin can reduce cerebral IR injury by inhibiting apoptosis and autophagy (40). Blocking hepatocyte PCSK9 improves HIRI by promoting PINK1-Parkin-mediated mitochondrial autophagy (41). Luteolin preconditioning inhibited inflammation, autophagy, and apoptosis through the ERK/PPAR pathway and alleviated liver IR injury in mice (42). In conclusion, most studies have shown that inhibition of autophagy can improve liver IR injury, which is consistent with our findings.

Studies have shown that Sch B regulates autophagy through multiple pathways. For example, Sch B induces ROS-mediated autophagy in Hepa1-6 cells via selenoproteins (43). In a hepatotoxic model, Sch B inhibits cell growth of mouse hepatocytes and macrophages, inducing apoptosis and autophagy (44). Schisandrin B improves oxidative stress and neuronal dysfunction induced by intracerebroventricular injection of amyloid by inhibiting RAGE/NF-kappaB/MAPK and upregulating HSP/Beclin expression (45). In addition to these signature genes, autophagy is regulated by many upstream genes, such as PI3K/Akt, MAPK, and mTOR signaling pathways (2). Galluzzi et al. found that when they pretreated the liver ischemia/reperfusion injury model with a caspase inhibitor, it could not improve the hepatic injury well, suggesting that not only apoptosis exists in the process of the injury. This suggests that apoptosis and other modes of cell death exist in this injury process (18). Further studies showed that autophagy is vital in liver IR injury. LC3-II protein is involved in the formation of autophagosomes in liver ischemia/reperfusion injury, suggesting that its expression can be interfered with to inhibit the formation of autophagosomes and ultimately reduce the ischemia/reperfusion injury. Various pretreatment modalities, such as ethyl pyruvate, hydrogen sulfide, and astaxanthin, have been found to down-regulate LC3-II expression, attenuate cellular autophagic activity after hepatic IRI, and attenuate liver injury. Therefore, cellular autophagy is an essential mode of cell death during the development of hepatic IRI and is an important target for future protection against hepatic IRI drug screening.

Our findings indicate that Sch B lowered liver enzyme levels and inflammation markers in a hepatic IRI experimental model. The protective function of Sch B against hepatic IRI may be associated with cellular autophagy.

To confirm our hypothesis, we extracted RNA from the liver tissues of mice treated with Fructus *Schisandrae* *chinensis*. We used qPCR to screen for mRNA level changes in key genes involved in HIRI pathogenesis, including HO-1, Nrf2, caspase-3, caspase-9, Bax, Beclin-1, and LC3-II. Beclin-1 and LC3-II mRNA levels were significantly reduced in the *Schisandra* berry ethanol group, with the most pronounced changes observed. Therefore, Fructus *Sch.* chinensis ethanol exerts its protective effect against hepatic IRI by inhibiting cellular autophagy. Rapamycin, a selective mTOR inhibitor, was used in this study as it is known to induce autophagy by inhibiting mTOR signaling. To detect the role of Sch B in protecting liver IRI by regulating autophagy, rapamycin was used to induce autophagy. The results showed that Sch B could significantly inhibit the occurrence of rapamycin-induced autophagy.

In conclusion, the present study demonstrated that Fructus *Sch.* chinensis possesses significant protective efficacy against hepatic IRI and confirmed that it exerts its protective effect by inhibiting cellular autophagy. This study examined the protective effects and mechanisms of Sch B on HIRI, offering insights into its clinical applications. The findings reveal the significant effect of Sch B in alleviating HIRI, providing a new option for the treatment of HIRI. Traditionally, treatments for HIRI have been limited by drug side effects, uncertain efficacy, or high costs. As a natural compound, Sch B may have better biocompatibility, lower toxicity, and a wider therapeutic window, and is expected to be an important supplement or alternative to HIRI therapy in the future.

The results of this study suggest that the severity of HIRI can be effectively alleviated by regulating cytoprotective mechanisms such as autophagy. This finding provides new ideas for optimizing existing treatment strategies. For example, Sch B can be combined with other drugs known to protect the liver to further improve the therapeutic effect through multi-pathway, multi-target synergies. Additionally, a personalized treatment plan can be developed according to the specific situation of the patient to maximize the protective effect of Sch B. This study not only provides a new perspective and tool for the basic research of HIRI but also provides a theoretical and experimental basis for clinical application. With further research and validation, we can explore the safety and efficacy of Sch B in humans, laying the foundation for translating it into an actual clinical treatment. This will help promote the close integration of basic research and clinical application, and advance the progress and development of HIRI therapy.

The results of this study may also inspire new research directions. For example, the mechanism of action of Sch B can be further explored to reveal its specific molecular pathways and targets in the regulation of cellular protection mechanisms such as autophagy. This will help us gain a deeper understanding of HIRI's pathophysiological processes and provide clues for the development of new therapeutic targets. Additionally, other natural compounds or drugs with similar protective effects can be explored to enrich the treatment of HIRI. Ultimately, the results of this study are expected to improve outcomes and quality of life for patients undergoing liver surgery or transplantation. By reducing the severity of HIRI and the incidence of complications, we can reduce patient mortality and length of hospital stay, improve surgical success, and liver transplant survival. This will help reduce the financial burden and psychological stress of patients and improve their quality of life and social participation.

In summary, the potential implications of the findings of this study for future HIRI treatment strategies are multifaceted and far-reaching. We look forward to translating this discovery into practical clinical applications for HIRI patients through further research and exploration.

There are some limitations in this study. The study of Sch B was only carried out in a mouse liver IR model, but further verification of the effect of Sch B on liver tissue injury by liver IR in rat or mammalian animal models or clinical trials will strengthen the conclusion about the efficacy and safety of Sch B in humans, which will be our team's future plan. Additionally, there are potential differences in the response of individual mice to Sch B treatment, and the sample size of Sch B in the liver IR model is not large. Long-term studies are needed to evaluate liver injury and chronic effects in the liver IR model, which will increase the rigor of the conclusions of this study.

Furthermore, there are many therapeutic methods for liver IR injury at present. Nobiletin improves liver IR injury by activating SIRT-1/FOXO3A-mediated autophagy and mitochondrial biogenesis (37). Dexmedetomidine mitigates ferroptosis after HIRI by upregulating Nrf2/GPX4-dependent antioxidant response (46). Arctiin ameliorates HIRI by reversing the aging fate of hepatic sinusoidal endothelial cells and restoring damaged sinus networks (47). The role of natural killer cells in HIRI has also been studied (48). Therefore, the combination of Sch B with other drugs or functional cells in the liver IR injury model will be a promising therapeutic strategy in the future.

### 5.1. Conclusions

Schisandrin B reduces liver cell damage by regulating autophagy, and its potential effect in reducing HIRI has been demonstrated in mouse models. This study provides a promising strategy for the prevention and treatment of HIRI, highlighting the potential therapeutic role of Sch B in improving liver surgery outcomes.

## Data Availability

All data can be requested or viewed from the corresponding author upon request.
